# Allosteric transition: a comparison of two models

**DOI:** 10.1186/2050-6511-14-4

**Published:** 2013-01-08

**Authors:** Niels Bindslev

**Affiliations:** 1 Synagics Lab, Endocrinology Section, Department of Biomedical Sciences, The Medical Faculty, Panum Building, University of Copenhagen, Blegdamsvej 3, DK-2200, Copenhagen N, Denmark

## Abstract

**Introduction:**

Two recent models are in use for analysis of allosteric drug action at receptor sites remote from orthosteric binding sites. One is an allosteric two-state mechanical model derived in 2000 by David Hall. The other is an extended operational model developed in 2007 by Arthur Christopoulos’s group. The models are valid in pharmacology, enzymology, transportology as well as several other fields of biology involving allosteric concentration effects.

**Results:**

I show here that Hall’s model for interactions between an orthoster, an alloster, and a receptive unit is the best choice of model both for simulation and analysis of allosteric concentration-responses at equilibrium or steady-state.

**Conclusions:**

As detailed knowledge of receptors systems becomes available, systems with several pathways and states and/ or more than two binding sites should be analysed by extended forms of the Hall model rather than for instance a Hill type exponentiation of terms as introduced in non-mechanistic (operational) model approaches; yielding semi-quantitative estimates of actual system parameters based on Hill’s unlikely simultaneity model for G protein-coupled receptors.

## Background

A sizeable decline in development of classical agonists and antagonist for medication [1-3] has elicited a drug-hunt to construct and develop allosters in laboratories of academia [4-8] and industry (e.g., Novasite Pharmaceuticals Inc; Addex Pharmaceuticals), including positive and negative allosters as well as ortho-allosters for therapeutic purposes. In doing so, it has become important to simulate and analyse concentration-response data for allosters by models that are as close to the systems mechanistic function as possible.

Optimal allosteric models are in great demand, since mechanistic simulations may be combined with structural analysis of alloster binding, receptor multi-merization and association of molecules as G proteins, arrestins, and RAMPs into synthesis of QSARs for ligand binding and receptor activation [9-16].

**Figure 1:**
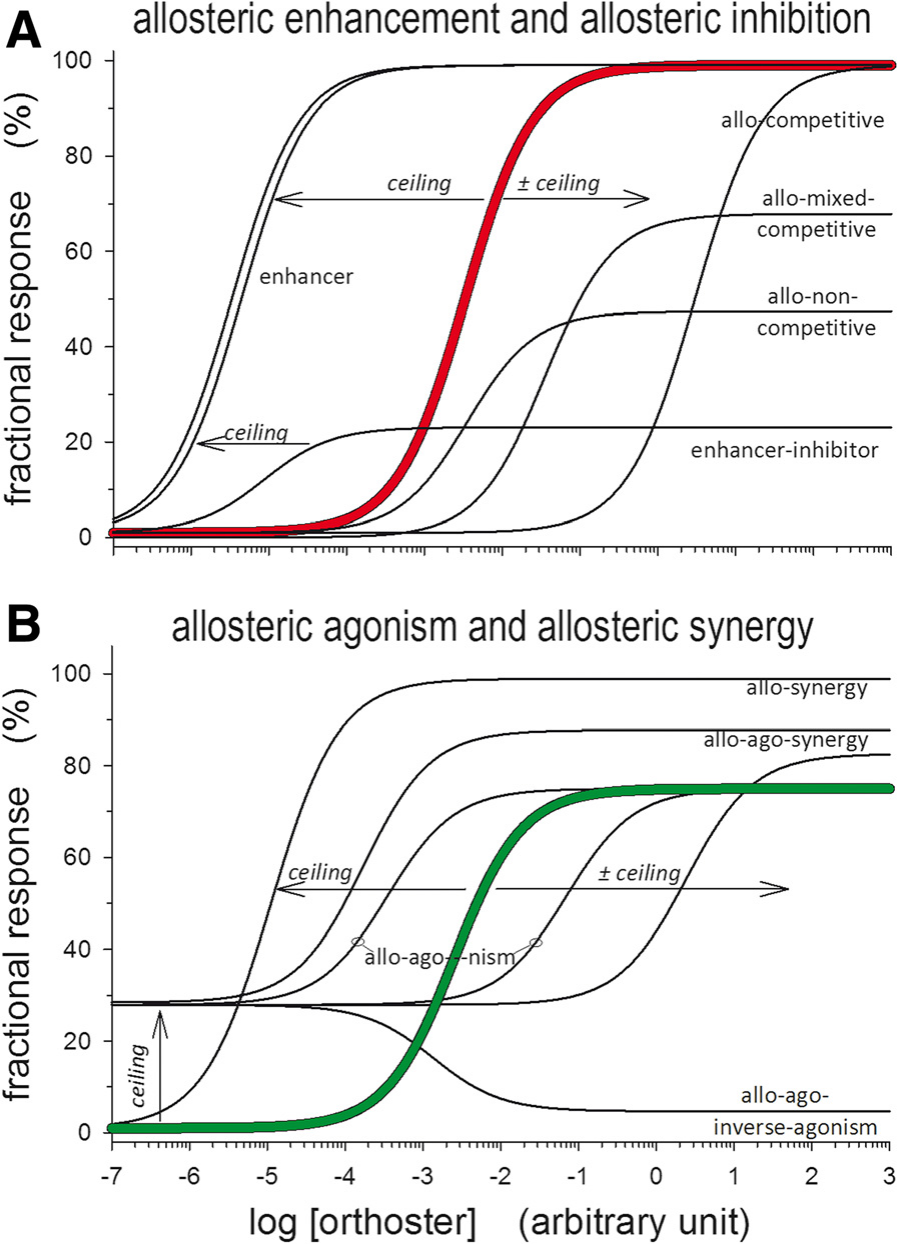
**Phenotypic behavior of allosters. Panel A.** Some concentration-response curves with an alloster present demonstrating enhancement and allo-inhibition of both a mixed and a competitive type antagonism and with ceiling effects for all three. The red curve represents an orthoster concentration-response in the absence of an alloster. **Panel B.** Concentration-response relations with an alloster present, displaying allo-agonism as a lifted initial activity with ceiling and allo-synergy as a lifted maximal response. Both allo-agonism and synergy curves are lifted compared to a concentration-response curve with no alloster present as in the green curve. Definitions of phenotypic alloster terms are listed in Table 1.

Data from equilibrium concentration-response experiments involving allosteric modulators are presently interpreted by unlike choices of model. Therefore, with such schism in selection of model, especially true for data from cell-systems expressing subtype 7TMRs [17], it seems worth a discussion about which direction analysis of synagics data for allosters should take. For possible outcomes of including allosters consult Figure 1. For definitions of terms related to allostery see Table 1.

**Table 1:** **Terms and definitions for allosteric synagics** (**see Figure**1)

**Term**	**Definition**
*orthoster*	primary ligand, binds at orthosteric (primary) receptor binding site and covers ligands as agonists, inverse agonists and (neutral) antagonists
*alloster or allosteric modulator*	secondary ligand, binds to a non-overlapping (secondary or allosteric) binding site distinct from an orthosteric binding site
*ago-alloster*	an alloster which can activate the receptor even in the absence of an orthoster, but with ceiling for the increased activity
*allo-agonism*	the effect of an ago-alloster
*syn-alloster*	alloster, at high orthoster concentrations it can still lift the response further with ceiling;
*allo-synergy* or *synergy*	the effect of syn-allosters, different from super-agonism
*ago-syn-alloster*	alloster, both activates receptors in absence of orthoster and increases activity even at high orthoster concentration. Both increases in activity have ceiling
*allo-ago-synergy*	the effect of ago-syn-allosters, different from super-agonism
*enhancer*	alloster, moves orthoster d-r curves to the left with ceiling
*allo-competitor*	alloster, moves orthoster d-r curves right with or without ceiling
*allo-mixed-competitor*	alloster, decreases activity and changes apparent affinity constants for orthosters. Orthoster d-r curves with allo-mixed-competitor are right-shifted but may have increased affinity
*enhancer-inhibitor*	alloster that both increases apparent affinity constants and decreases activity for orthosters. With enhancer-inhibitor, orthoster d-r curves move left with ceiling
*ago-inverse-alloster*	alloster, stimulates activity from an allosteric site in its own right, but with an activity which is reduced with increasing orthoster concentrations
*ortho-alloster* or *bitopic ligand*	compound with moieties for simultaneous binding and activation at both orthosteric and allosteric receptor binding sites
*synagics*	the study of equilibrium and steady-state concentration-responses of ligand interactions with receptive units such as protein macromolecules
*positive and negative allosteric modulators*	(PAMs* and NAMs**) - ligands that increase or decrease receptor activity directly or indirectly from an allosteric binding site.

*PAMs cover both ago-allosters, syn-allosters, and ago-syn-allosters. Enhancers may be included here. ** NAMs cover both allo-mixed-competitors, enhancer-inhibitors, and ago-inverse-allosters. Allo-competitors may be included here.

*Two actual allosteric models* - *ATSM and EXOM*. One model is the allosteric two-state model, ATSM, introduced by Hall in 2000, implemented and further discussed by others [5,17-25]. Another model we could call the “extended operational model”, EXOM for short [26], is based on combining the original operational model, BLM [27], with the ternary-complex model, TCM [28], as later further detailed [29-31]. EXOM is implemented and presently advocated by several lead-modellers [7,8,32-38]. There are other approaches taken to model the behaviour of allosters in the field of 7TMRs [20,33,39-42].

ATSM is a mechanistic model. ATSM-analysis with extracted numbers for model parameters supposes direct information about mechanical interactions between allosters, receptors and orthosters at a molecular scale. Thus, one might gain a quantitative and dynamic handle on molecular processes *per se* within receptors. The other model, EXOM, a non-mechanistic model, is a close relative of ATSM and has the same number of independent parameters to be determined. EXOM is used assuming that individual physical parameters of multi-step processes as such cannot be extracted, as they are composite. EXOM may give quantified estimates on elicited cooperative binding and efficacy for orthosters and allosters interacting at receptors [26,34]. By selecting similar assumptions for ATSM as for EXOM, ATSM may cover the EXOM-scenario and yield estimates of parameters for lumped multi-steps rather than single steps, and thus become a black-box model as the EXOM.

In both ATSM and EXOM, allosters may behave as enhancers with ceiling and as competitive antagonists without ceiling. Furthermore, they are also efficient in simulating allo-agonism and allo-synergy both with ceiling effects; observed as lifts of concentration-response curves by allosters at low and high orthoster concentrations [17,26,37]. However, EXOM lacks ATSM’s advantage of being a mechanistic model and for describing spontaneous activity of receptive units. Additionally, from a theoretical point of view, a parameter in EXOM to describe cooperative activity is amputated, yielding illogic results. For this latter conclusion, see details in the next to last sections of Methods and Results and Discussion.

Here I focus on ATSM and EXOM and compare them for simulation and analysis of experimental data. It is demonstrated that there are no arguments as posited [8,17] for employing EXOM instead of ATSM, quite the other way about. Therefore, my goal is to convince future modellers to use ATSM and possible extended forms for analysis and simulation of allosteric concentration-response relations rather than EXOM.

## Methods

### One basic model - cTSM

In simulation of synagics for orthosters and allosters, the basis of most models is often two simple reaction schemes; the cyclic-two-state model, cTSM, and the ternary-complex model, TCM. Since this paper is about modelling as opposed to general statements about ligand-receptor interactions it is paramount with precise definitions including aspects of cTSM and TCM. This has been discussed before [22] and may seem superfluous. However, in order to validate and compare newly derived ATSM and EXOM in a coherent fashion, concepts related to cTSM and TCM must be brought together and systematized. cTSM is dealt with first.

**Figure 2:**
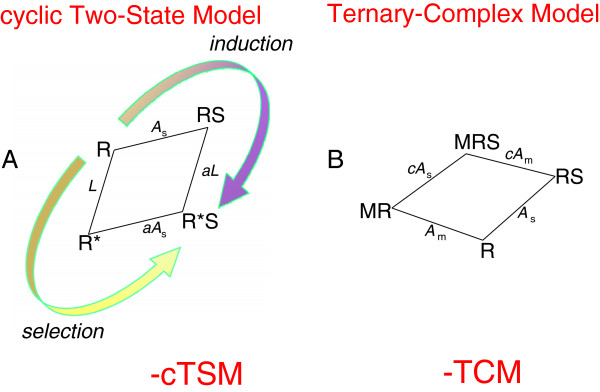
**Two simple reaction schemes. Panel A.** The cyclic two-state model, cTSM, with selection and induction arrows indicating two separate but simultaneous pathways from an inactive and non-liganded receptor conformation R to an active and agonist S liganded receptor conformation R*S. *A*_s_ is an equilibrium association constant for S, *L* is a conformational efficacy constant for non-bound receptors, and parameter *a* is an efficacy constant for ligand bound receptor conformations from RS to R*S. **Panel B.** The ternary-complex model, TCM, in which symbol M represents the term and concentration for an additional alloster ligand. *A*_m_ is an equilibrium association constant for M, and parameter *c* is a cooperativity coefficient for two-ligand binding.

The gist of the cTSM, Figure 2A, is its explicit description of a conformational switch between an inactive and active state of a non-bound receptor. It specifically includes spontaneous activity in form of non-liganded receptor R*. The behaviour of cTSM has been scrutinized [43,44]. cTSM has two interesting parameters. *L* describes the distribution between unliganded inactive and active receptor states, R ⇌ R*, such that *L* = R*/R, Figure 2A. Deriving cTSM’s distribution equation for activity, the free non-active receptor state R is equated with “1”. Thus, the unliganded, active receptor state R* is equal to *L*. The second parameter, *a*, is a concomitant constant for activation of receptor forms bound with ligand S, RS ⇌ R*S. This step has *a*·*L* as its efficacy constant. By assuming multi-steps, *a*·*L* is identical to Stephenson's efficacy constant [45] and Black & Leff’s transducer ratio *τ*[27]. *A*_s_ is the equilibrium affinity constant for S binding to non-active forms of R, Figure 2A. Therefore, *a* is also a concomitant constant for binding of S to already activated receptors. The affinity constant for S+R* ⇌ R*S is thus *a*·*A*_s_.

Arguments still appear on how to understand activation of protein molecules when ligands are applied - is it by *induction* after ligands bind or is it rather by ligand *selection* and stabilization of already activated molecules? Jacques Monod early on favoured a selection process [46] and this understanding crystallized in the famous MWC-model [47]. The MWC explicitly introduces an unliganded switch R⇌R* as the “allosteric transition” [48]. Contrary, Koshland argued for induction after binding [49]. “Selection” follows one leg of cTSM while “induction” follows another [50], Figure 2A. They are two views on a single process [18] chapter 5. Below, when either “induction” or “selection” is used on activation of receptive units as ligands bind, it covers both pathways in cTSM.

### Another basic model - TCM

The TCM, Figure 2B, looks fairly simple, but possesses surprising allosteric regimes. Depending on which of the liganded complexes are included for activity, TCM can simulate enhancement with ceiling and competitive (“surmountable”) inhibition, besides allo-agonism without ceiling and “mixed competitive inhibition”. TCM with tacit active conformations has no allo-synergy or spontaneous activity. Ten sub-models derived from TCM are characterized in Table 2. Three of these sub-models are further described in the Results section and some simulations by these three models are shown in a figure in the Results section.

**Figure 3:**
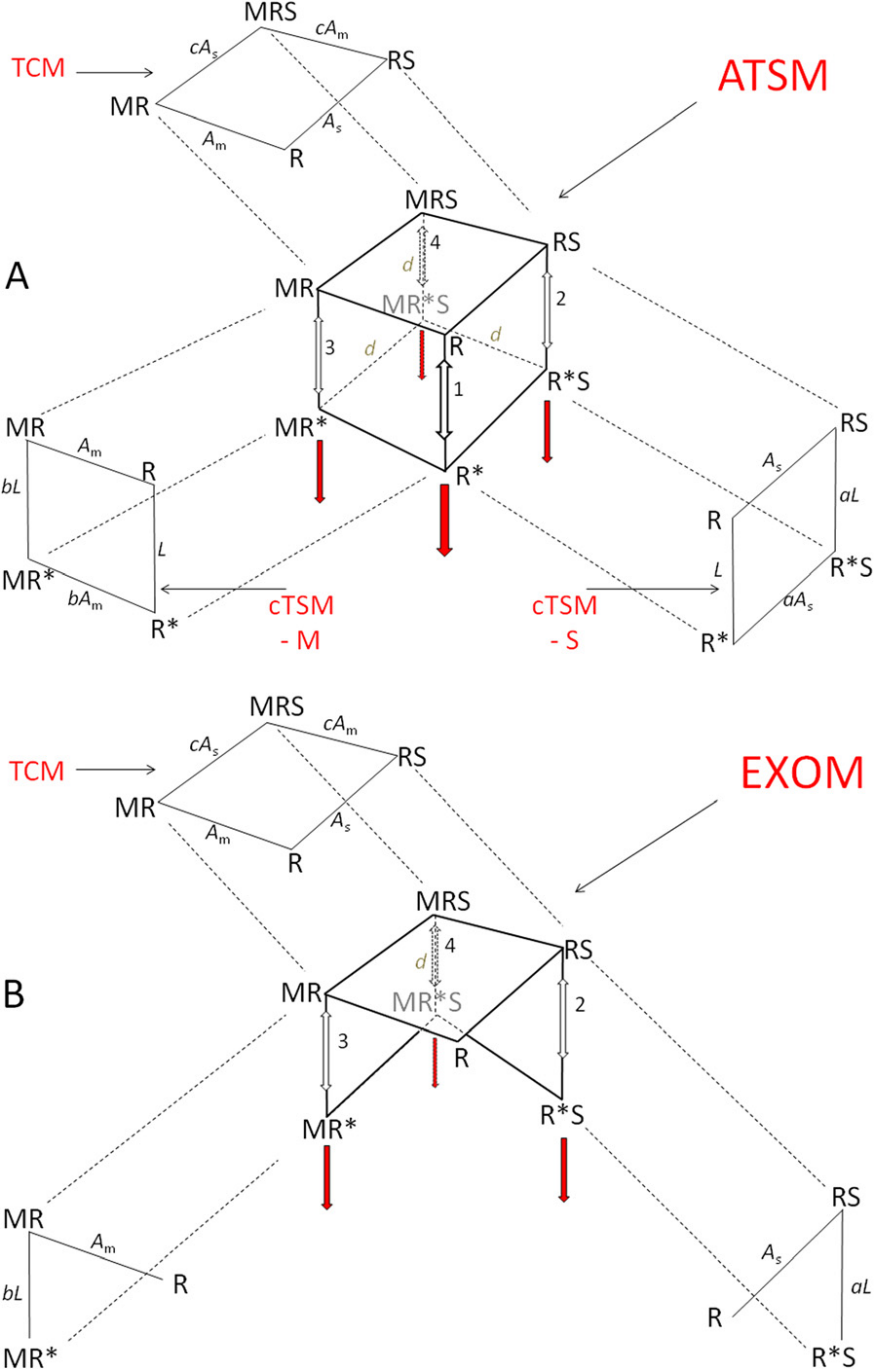
**Simulations form four sub-models of the ternary-complex model, TCM.** For sub-model definitions see Table 2. Parameters *A*_s_ and *A*_m_, equilibrium association constants for ligands S and M, are kept at unity. Parameter *c*, the cooperativity constant for binding, is varied by a factor 10^3^ in three steps for each sub-model as indicated in the panels. Red curves indicate orthoster concentration-response curves in the absence of an alloster. In all panels the alloster M concentration is varied in four steps: in panels A-I by a factor 10^2^ from 1x10^-2^ to 1x10^4^; in panels G-K by a factor 10 from 1x10^-2^ to 1x10^1^ and in panel L by a factor 10^2^ from 1x10^-3^ to 1x10^3^. Green curves with circles show the actual EC_50_ and the black circle represents the position of a limiting EC_50_ for M → ∞.

**Table 2:** **Phenotypic concentration**-**responses for allosters in 10 sub**-**models from TCM**

***Type of TCM model***	**#**	***Enhancement*** ←	***w***/ ***ceiling*** ←	***Allo***-***agonism*** ↑	***w***/ ***ceiling*** ↑	***Strict allo***-***synergy*** ↑	***Allo***-***modification w***/ ***ceiling*** → ↓
(S)/4	1	no	na	no	na	no	modifier - EC_50_ ↓
(S+MS)/4	2	yes	yes	no	na	no	competitive
(S+M+MS)/4	3	yes	yes	yes	no	no	na
(S+M)/4	4	no	na	inverse	yes	no	yes
(MS)/4	5	(yes)	yes	no	na	(yes)	na
(S)/3	6	no	na	no	na	no	modifier - EC_50_ ↓
(S+MS)/3	7	yes	→	no	na	no	no
(MS)/3	8	(yes)	no	no	na	(yes)	no
(S)/3*	9	no	na	no	na	no	competitive**
(S+M)/3*	10	no	na	yes	no	no	no

For model types in the left column, terms S, M, and MS in parenthesis indicate active forms of the liganded receptor as either R*S, M*R or MR*S, and with the total number of receptor conformations after the slash. In models 6–8, complex MR is not formed. Model 7 is the classical uncompetitive reaction scheme. * In models 9–10, complex MRS is not formed. **Model 9 is classical type II reaction scheme for competitive inhibition with no ceiling, the same as assuming parameter *c* = 0. Arrows indicate direction of affinity change and direction of ceiling effects.

na = not applicable, (yes) indicates that there is an effect in form of co-agonism, i.e., no response for ligand S alone.

**Figure 4:**
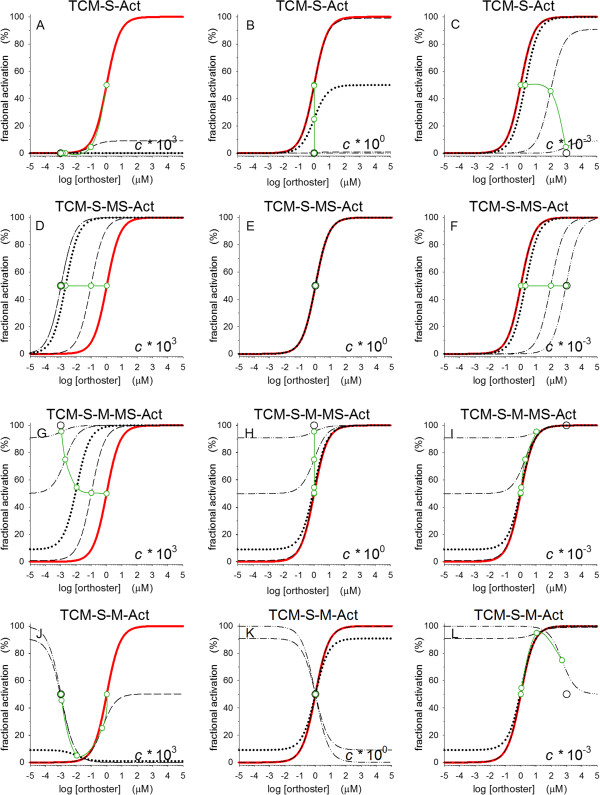
**Reaction schemes of the allosteric two-state model, ATSM, and the extended operational model, EXOM. Panel A.** The ATSM. **Panel B.** The EXOM. The models are presented with their basic simpler reactions schemes as the cTSM and TCM from Figure 2. The cubic ATSM has eight receptor conformations while the EXOM only has seven of those, as the spontaneous active represented by receptor conformation R* is excluded. The two models have the same total number of parameters, seven in all. Besides parameters defined in Figure 2, ATSM and EXOM have parameter *b*, an efficacy constant when the alloster M-bound receptor is activated, and parameter *d* a cooperativity efficacy constant involving two ligands. The constants *L*, *A*_s_, *A*_m_ , *a* and *c* are given as in Figure 2, and EXOM has a slope factor *n*, not shown.

**Figure 5:**
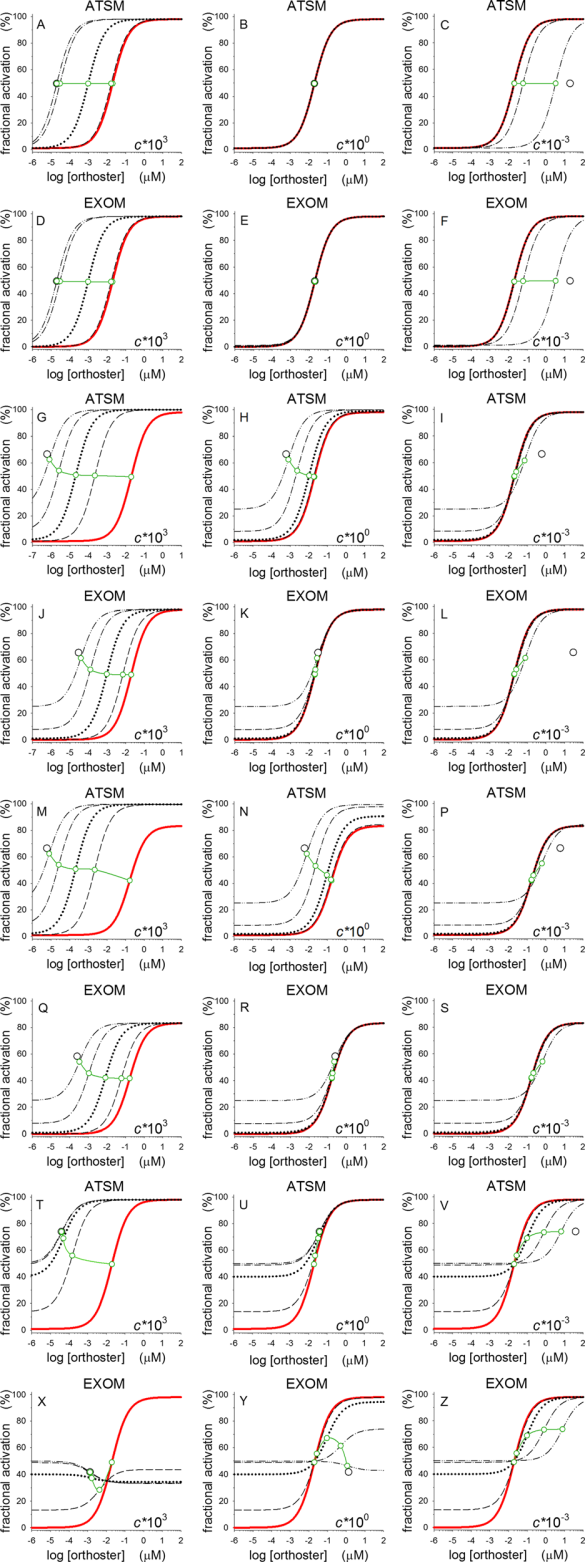
**Simulations of concentration-response relations for ATSM and EXOM.** The parameters *A*_s_ and *A*_m_ are both kept at unity, while parameter *L* is 10^-2^ for all ATSM simulations in order to keep spontaneous activity insignificant and *n* for all EXOM simulations is = 1. Parameter *c*, the binding cooperativity constant, is varied in three steps by multiplying with a factor 10^3^ from 10^-3^ to 10^3^ as indicated in the panels. Parameter *a* is 5000 in all ATSM panels except for panels M-P where it is 500. For EXOM, parameter *a* is 50 in all panels except for panels Q-S where it is 5. For ATSM, parameter *b* is 1 in panels A-C, and 50 in the rest of panels G-V. For EXOM, parameter *b* is 0.01 in panels D-F, and 0.5 in the rest of panels J-Z. Parameter *d* is 1 in all panels except in panels T-Z where it is 3x10^-3^. All red curves have no alloster present, i.e., concentration of M = 0. M is varied in four steps. In panels A-F by a factor 100 from 2x10^-4^ to 2x10^2^; in panels G-S by a factor 10 from 2x10^-3^ to 2x10^0^; and in panels T-Z by a factor 10 from 2x10^-1^ to 2x10^2^. Green curves with circles show the actual EC_50_ and the black circle represents the position of a limiting EC_50_ for M → ∞. The black circle falls outside the orthoster concentration range, 10^-6^ to 10^2^, in panels S and Z with limiting EC_50_ values of 250 and 1304.

Simulations of concentration-response relations for tabulated sub-models 1–4, in column 2, are shown in Figure 4 panels A-I. S stands for orthoster and M for alloster. Ceiling effects for enhancement (= parameter *c* > 1) in sub-model 2 starts at *A*_m_· M > 1, panel D in Figure 4. Allo-competitive antagonism (= parameter *c* < 1) in sub-model 2 requires *c*·*A*_m_·M > 10 for a ceiling effect to appear. Thus, sub-model 2 simulates genuine competitive antagonism as long as the product *c*·*A*_m_·M is below 10, Figure 4 panel F. This dependence on product *A*_m_·M > 1 for ceiling effects of enhancement and on product *c*·*A*_m_·M >10 for ceiling effects in allo-competitive inhibition are also characteristics of both ATSM, Figure 5 panels A and C, and EXOM, Figure 5 panels D and F.

Tabulated ternary-complex sub-model 1 and 6 with parameter *c* < 1 are characterized as (*mixed*) *modifier mechanisms* in enzymology. Their mixed allo-modification includes a possible simulation of classical non-competitive antagonism with a fixed EC_50_, when *c* = 1, Figure 4 panel B. Furthermore, both sub-models 1 and 6 have increasing affinity for increasing modifier concentration, indicated by EC_50_ ↓ in column 8. Sub-type model 4, excluding the ternary complex MRS as active, may show inverse agonism with decreasing ceiling values for the apparent affinity EC_50_ when parameter *c* > 1 and increasing ceiling levels for EC_50_ when parameter *c* < 1, Figure 4 panels J-L.

Sub-models 5 and 8 demonstrate co-agonism, which means that both ligand S and ligand M have to be present for an activity to show up, simulations not shown.

Sub-model 7 is identical to the classical un-competitive reaction scheme. Sub-models 9 and 10 are based on the classical type II competitive reaction scheme, excluding the double-liganded MRS conformation ([18] chapter 2), and therefore do not qualify as true TCMs.

Two characteristics for ATSM and EXOM are not covered by any of the listed TCM reaction schemes in Table 2, *viz*. a strict allo-synergy, Figure 5 panels M and N, and ceiling effects for allo-agonism, compare Figure 4 panels G-I with Figure 5 panels G-H, J-K, M-N, Q-R, and T-U.

### Operational models

To understand the present use of “stimulus”, “efficacy” and “intrinsic efficacy” in operational models as EXOM, it is necessary to go back to their definitions [45,51,52]. Stephenson’s stimulus concept seems obsolete today by accepting two-step receptor schemes with straightforward derived distribution equations [18] chapter 2; [50] and when needed, apt assumptions of more than two steps. Two-step schemes yield equations identical to initially derived operational models based on the stimulus-response idea [27,51,53]. Concepts as “stimulus”, “transducer ratio” and “fitting parameter” are of course justified in selecting operational model approaches rather than mechanistic ones. Spontaneous activity often seen in studies with 7TMRs is not included in the realm of operational models, although recently serious attempts have appeared [54,55].

Meanwhile, users of operational models should recognize that their assumptions for derivation put a veil over underlying physical systems and that any involved “operational” assumption may just as well be applied to the ATSM. For instance, as mentioned, *a*·*L* can be conceived as equal to transducer ratio *τ*.

### Distribution equation for ATSM and EXOM

Reaction schemes of ATSM and EXOM are depicted in Figure 3A and 3B. The intention with EXOM was to derive a stimulus-equation for activating receptors, including alloster-activated units, while explicitly excluding non-liganded active conformations [26]. Thus, three bound species RS, MR, and MRS in EXOM can switch to active forms R*S, MR*, and MR*S. But, in order to exclude constitutive activity, non-liganded R is not allowed a switch to active R*, Figure 3B. Thus, EXOM is a pure “induction” reaction scheme in Koshland-sense, as free forms of receptor R must be bound before activation. The three bound and active forms of the receptor are equated as “stimulus” and transformed through a hyperbolic expression for activity, as for the BLM. The result is a distribution equation with three active conformations to a total of seven conformation, as even a possible inactive R*-conformation is considered non-existent [26].

To simplify a comparison of EXOM with ATSM, distribution equations for both are expressed parallel to earlier expressions for ATSM [18] chapter 7.

This yields for activity in EXOM:

$$E=\frac{E_{m}\cdot {\left[a\cdot A_{s}\cdot S+b\cdot A_{m}\cdot M+a\cdot c\cdot d\cdot A_{s}\cdot S\cdot A_{m}\cdot M\right]}^{\left(n\right)}}{{\left[1+A_{s}\cdot S+A_{m}\cdot M+c\cdot A_{s}\cdot S\cdot A_{m}\cdot M\right]}^{\left(n\right)}+{\left[a\cdot A_{s}\cdot S+b\cdot A_{m}\cdot M+a\cdot c\cdot d\cdot A_{s}\cdot S\cdot A_{m}\cdot M\right]}^{\left(n\right)}}$$

and for activity in ATSM:

$$E=\frac{E_{m}\cdot \left(L\cdot \right)\left[\left(1+\right)a\cdot A_{s}\cdot S+b\cdot A_{m}\cdot M+a\cdot \left(b\cdot \right)c\cdot d\cdot A_{s}\cdot S\cdot A_{m}\cdot M\right]}{1+A_{s}\cdot S+A_{m}\cdot M+c\cdot A_{s}\cdot S\cdot A_{m}\cdot M+\left(L\cdot \right)\left[\left(1+\right)a\cdot A_{s}\cdot S+b\cdot A_{m}\cdot M+a\cdot \left(b\cdot \right)c\cdot d\cdot A_{s}\cdot S\cdot A_{m}\cdot M\right]}$$

Deviations between the two models are marked by bracketed and bolded symbols. Definitions of symbols listed below are followed by symbols in parenthesis from Leach [26] and Hall [22]: E = actual response; E_m_ = maximal activity; S = orthoster (A; A); M = alloster (B; B); *A*_s_ = equilibrium association constant for ligand S (1/*K*_A_ , *K*); *A*_m_ = equilibrium association constant for ligand M (1/*K*_B_, *M* ); *a* = efficacy constant for S (*τ*_A_; *α*); *b* = efficacy constant for M (*τ*_B_; *β*); *c* = binding cooperativity constant (*α*; *γ*); and *d* = activation cooperativity constant (*β*; *δ*). Parameter *β* for EXOM is only defined for cooperativity of an alloster on orthoster activation, but not reciprocally as in ATSM. Further, unlike ATSM, EXOM has a Hill type exponentiation parameter, *n*, for terms of summed activity and inactivity. The benefits of including such a Hill exponentiation may be questioned as discussed earlier [18] chapter 10. Indeed, Hill-type exponentiation may also be applied to ATSM. However, as ATSM is a mechanistic approach, it seems more logical to derive equations based on formulation for an extended ATSM with more than two binding sites [18,25].

In absence of an orthoster the initial efficacy, IntEff, for ATSM is given by: ***L***/[***L***+(1+ *A*_m_·M)/ (1+*b*·*A*_m_·M)], and for EXOM, assuming *n* = 1, by: **1**/[**1**+(1+*A*_m_·M)/(1+*b*·*A*_m_·M)].

For high values of the orthoster, S⇒∞, maximum activity, MaxEff, as a function of alloster concentration for ATSM is given by: ***L***/[***L*** + (1 + *c* · *A*_m_ · M)/(*a* · (1 + ***b*** · *c* · *d* · *A*_m_ · M))], and for EXOM, assuming *n* = 1, by: **1**/[**1** + (1 + *c* · *A*_m_ · M)/(*a* · (1 + *c* · *d* · *A*_m_ · M))]. Differences between ATSM and EXOM expressions are indicated with bolded types.

### Best-fit analyses to experimental data for ATSM and EXOM

The analyses were performed in the following manner. Selected allosteric effects were obtained from data-figures in the literature, data-figure 1 ([38], Figure 2B), data-figure 2 ([37], Figure 2B), and data-figure 3 ([56], Figure 3). Model parameters *a* and *A*_s_ were first evaluated by fitting the distribution equations for ATSM and EXOM to response data at zero alloster concentration. The obtained values for *a* and *A*_s_ were then inserted into the distribution functions for the two models and used for an ensuing fitting of the remaining parameters listed in the last Table, parameters *b*, *c*, *d*, and *A*_m_. By varying the initial values for each parameter in three steps, at least 12 fits were performed on each curve for every alloster concentration in all three data-figures. Only fitted parameter values with convergence to a tolerance of 10^-10^ in SigmaPlot software were accepted.

Thus, concentration-response curves at three different alloster concentrations yields three best-fit values for each of the four parameters. Obtained results for the single parameter in the last Table represent a ratio between the two best-fit values with the largest mutual difference of the three determinations for each parameter at different alloster concentrations. A global fit to data sets for all four parameters [57] was not possible.

A fourth data set, data-figure 4 ([36], Figure 1C), was also analysed but neither ATSM nor EXOM fitted well to these data with a 44% spontaneous activity and a 56% alloster/ orthoster response. The failure of fitting was mostly due to a lack in obtaining a reasonable determination of maximal response for several of the concentration-response curves.

## Results and discussion

### TCM - three and ten variants

Three functional variants of TCM are briefly described below and examples of their simulations shown in Figure 4, while characteristics of ten different forms derived from TCM are listed in annotated Table 2.

In a first form, complex RS tacitly moves to R*S as the sole source of activity. Simulation of this allo-scheme can resemble classical non-competitive antagonism for orthosters in functional assays, where only the maximal effect attenuates as the concentration of an alloster increases while the dissociation constant for the agonist stays constant. This happens for activity when constant *c* is unity. An example is shown in Figure 4B. Note, that in TCM occupancy, alloster effects can never be non-competitive-like, i.e., with reduced activity and fixed EC_50_.

In a second form, S-liganded conformations, RS and MRS, move tacitly to R*S and MR*S as source of activity. This reaction scheme gives us models of activity and occupancy that behave in an identical manner as their distribution equations are identical. This reaction scheme includes enhancement for constant *c* > 1 and with ceiling when *A*_m_·M > 1 and competitive inhibition when *c* < 1, but with a ceiling effects for both binding and activation by an alloster when *c*·*A*_m_·M > 10, Figure 4D and 4F. This model is identical to the uncompetitive reaction scheme.

In a third form, all liganded conformations, i.e., RS, MR, and MRS, are sources of activity, Figure 2B. In EXOM, this is the basic TCM. TCM sub-type 3 may simulate allo-agonism for activity, but without ceiling effects as indicated by black circles for limiting EC_50_ values as M → ∞, Figure 4G-I.

Since the term “competitive inhibition”, according to an informative review [48], meant inhibition through an overlap or steric hindrance at binding sites [58], the term “allosteric inhibition” was used from the start of the 1960s merely to indicate negative feedback different from competitive inhibition. Nothing more. TCM with its two remote binding sites has no mutual exclusion by steric hindrance or by overlap. Meanwhile, TCM may still simulate “competitive inhibition”, either by its uncompetitive form as shown in Figure 3F, or by mutual exclusion of triple complex MRS through remote or intermolecular conformational changes, not shown. Thus, TCM has allosteric inhibition in the MWC-sense. “Competitive inhibition” by mutual exclusion in TCM requires that the cooperative binding constant *c* goes to insignificantly small values, thus preventing detectable levels of MRS and of its tacitly active form, MR*S. Such allosteric mutual exclusion, as one type II competitive inhibition ([18], chapter 2) has been cartooned ([58], Figure III-1, panel 5). Thus, as “allosteric” solely refer to ligand binding at remote, non-overlapping binding sites and without steric hindrance, “allosteric” becomes a pleonasm in “allosteric ternary complex model”, ATCM, as TCM is defined by having two, non-overlapping binding sites without steric hindrance. As both acronyms cover the exact same model, it remains a matter of taste using either ATCM or TCM. Contrary, the signifier “allosteric” in “allosteric transition” [48] becomes indicative for two-state models as MWC and ATSM, involving cTSM.

### Comparison of simulations from ATSM and EXOM

A comparison is made between ATSM and EXOM simulations of concentration-responses of activity with orthoster concentration as independent variable and with varying alloster concentration M. Thus, the following are principal statements about parameter influences on initial and maximal efficacies, on ceiling effects for enhancement, competitive and mixed inhibition, on allo-agonism and -synergy, as well as on apparent dissociation constant EC_50_. To simplify the comparison, EXOM slope factor *n* is assumed unity. The results reveal a few crucial differences between the two models even based on homologous parameters as *A*_s_, *A*_m_, *a*, *c*, and *d*.

As indicated above, IntEff for EXOM is dependent on parameter *b*, while for ATSM it is dependent on both *b* and *L*. For ATSM, MaxEff is dependent on *L*·*a*, whilst EXOM-MaxEff is only dependent on *a*. Thus, when comparing ATSM and EXOM, choice of values for *a* and *b* in EXOM should match with values for *L*·*a* and *L*·*b* in ATSM. Accordingly, in selection of parameter values for compared simulations with *L* for ATSM chosen as 0.01 in order to suppress spontaneous activity, values for *a* and *b* in EXOM are chosen 100 fold higher in ATSM, Figure 5.

IntEffs for both ATSM and EXOM are always completely independent of *A*_s_, *a*, *c*, and *d*. ATSM-IntEff is dependent on *L* and *b*·*A*_m_·M. For more details see annotated Table 3. EXOM-IntEff only depends on *b*·*A*_m_·M. Allo-agonism is a lift in the IntEff when supplying an alloster even before an orthoster is added. Various forms of allo-agonism are shown in Figure 5G-Z and with ceiling effects indicated by black circles for the limiting EC_50_ values as M → ∞. Allo-agonism is often seen in studies with small molecule allosters [59]. Allo-agonism takes effect in both models when both *b* and *b*·*A*_m_·M are larger than unity. Furthermore, ATSM may simulate spontaneous activity before any ligand is added. Simulation of detectable spontaneous activity starts at values of *L* above 10^-2^. This possibility is excluded from the EXOM theory.

**Table 3:** Conditions for alloster effects on initial efficacy and maximal efficacy in ATSM

***Assumptions for product***	***Reduced equation***	***Lower level product assumptions***	***Reduced equation at lower level product assumptions***	***IntEff / MaxEff, their dependence on product of***
***parameters ·[M]***				
*possible allo*-*agonism of****IntEff****for* [*orthoster*] → *0*: *L*/[*L*+((1+*A*_m_· M)/(1+*b*·*A*_m_· M))]				
*b*·*A*_m_· M >> 1	*L*·*b*·*A*_m_·M = X	*A*_m_·M >> 1	*L*·*b* /(*L*·*b* +1)	*L*·*b* vs 1
	X/(X+1+*A*_m_· M)			
		*A*_m_·M = 1	*L*·*b* /(*L*·*b* +2)	*L*·*b* vs 2
		*A*_m_·M << 1	*L*·*b* /(*L*·*b* +1)	*L*·*b* vs 1
*b*·*A*_m_· M = 1	*L*·2/(*L*·2+1+*A*_m_· M)	*A*_m_· M >> 1	*L*·2/(*L*·2+*A*_m_· M)	*L*·2 vs *A*_m_· M
		*A*_m_· M = 1	*L*/(*L*+1)	*L* vs 1
		*A*_m_· M << 1	*L*·2/(*L*·2+1)	*L*·2 vs 1
*b*·*A*_m_· M << 1	*L*/(*L*+1+*A*_m_·M)	*A*_m_· M >> 1	*L*/(*L*+*A*_m_· M)	*L* vs *A*_m_· M
		*A*_m_· M = 1	*L*/(*L*+2)	*L* vs 2
		*A*_m_· M << 1	*L*/(*L*+1)	*L* vs 1
*possible allo*-*synergy of****MaxEff****for* [*orthoster*] →∞ : *L*·*a*/[*L*·*a*+((1+*c*·*A*_m_·M)/(1+*b*·*c*·*d*·*A*_m_·M))]				
*b*·*c*·*d*·*A*_m_·M >> 1	with *L*·*a*·*b*·*c*·*d*·*A*_m_·M =Y	*c*·*A*_m_·M >> 1	*L*·*a*·*b*·*d*/(*L*·*a*·*b*·*d*+1)	*L*·*a*·*b*·*d* vs 1
	Y/(Y+1+*c*·*A*_m_·M)	*c*·*A*_m_·M = 1	*L*·*a*·*b*·*d*/(*L*·*a*·*b*·*d*+2)	*L*·*a*·*b*·*d* vs 2
				*b*·*d* >> 1
		*c*·*A*_m_·M << 1	Y/(Y+1)	Y vs 1
				*b*·*d* >>> 1
*b*·*c*·*d*·*A*_m_·M = 1	with *L*·*a*·2 = Z	*c*·*A*_m_·M >> 1	Z/(Z+*c*·*A*_m_·M)	Z vs *c*·*A*_m_·M
	Z/(Z+1+*c*·*A*_m_·M)	*c*·*A*_m_·M = 1	*L*·*a*/(*L*·*a*+1)	*L*·*a* vs 1
				*b*·*d* = 1
		*c*·*A*_m_·M << 1	*L*·*a*·2/(*L*·*a*·2+1)	*L*·*a*·2 vs 1
*b*·*c*·*d*·*A*_m_·M << 1	*L*·*a*/(*L*·*a*+1+*c*·*A*_m_·M)	*c*·*A*_m_·M >> 1	*L*·*a*/(*L*·*a*+*c*·*A*_m_·M)	*L*·*a vs c*·*A*_m_·M
		*c*·*A*_m_·M = 1	*L*·*a*/(*L*·*a*+2)	*L*·*a* vs 2
				*b*·*d* >> 1
		*c*·*A*_m_·M << 1	*L*·*a*·/(*L*·*a*+1)	*L*·*a* vs 1

Initial and maximal response for ATSM with orthoster concentration as independent variable with an interfering alloster. M or [M] stands for alloster concentration. Conditions are listed with decreasing number of parameters from column 1 to 5 for products of M and parameters that affect the initial efficacy, IntEff, at very low concentrations of orthoster, S, and the final maximal efficacy, MaxEff, at very high concentrations of S.

All conclusions for IntEff and MaxEff of ATSM are similar for the EXOM with the following exceptions: for EXOM 1) parameter *L* is replaced with 1in all statements for ATSM and 2) parameter *b* disappears out of all MaxEff statements as listed for ATSM.

Below are further details about effects of parameters and alloster concentration on IntEff and MaxEff for ATSM and EXOM.

***Initial efficacy***. ***IntEff****for ATSM or spontaneous activity*:

For *b* = 1, IntEff = *L*/(*L*+1) and independent of the value of *A*_m_·M.

For *b* > 1, IntEff >*L*/(*L*+1). With increasing values of *A*_m_·M above 1 the IntEff increases towards a ceiling value of *L*·*b*/(*L*·*b*+1), equal allo-agonism. For decreasing values of *A*_m_·M below 1, the IntEff goes towards *L*/(*L*+1).

For *b* < 1, IntEff <*L*/(*L*+1). With increasing values of *A*_m_·M above 1 the IntEff reduces towards a ceiling value of *L*·*b*/(*L*·*b*+1). For decreasing values of *A*_m_·M below 1, the IntEff increases towards *L*/(*L*+1).

Allo-agonism above spontaneous activity in ATSM, *L*/(*L*+1), is given by *L*·*b*/[*L*·*b*+1+1/(*A*_m_·M)], when both *b*·*A*_m_·M >> 1 and also parameter *b* > 1. The ceiling value of this allo-agonism is *L*·*b*/(*L*·*b*+#), where # is a value between 1 or 2, depending on the value of *A*_m_·M.

***IntEff****for EXOM*:

Allo-agonism in EXOM is always given by *b*/[*b*+1+1/(*A*_m_·M)], and going towards zero for *b* → 0, independent of the value for *b*·*A*_m_·M, and with a ceiling level of *b*/[*b*+¤], where ¤ is a value between 1 or 2, depending on the value of *A*_m_·M. Examples of ceiling effects and their absence in ATSM and EXOM are shown in Figure 5. For 1/(*A*_m_·M) >>*b*+1 in EXOM, IntEff goes towards 0 if *b* < 1, while for 1/(*A*_m_·M) <<*b*+1, IntEff approaches *b*/(*b*+1) as its ceiling level.

***Maximal efficacy***. ***MaxEff****for ATSM*:

When *b*·*c*·*d*·*A*_m_·M >> 1 and as long as *c*·*A*_m_·M ≥ 1, ATSM-MaxEff is always dependent on the product *b*·*d* and independent of the value of *c*·*A*_m_·M.

For *b*·*d* = 1, MaxEff = *L*·*a*/(*L*·*a*+1), independent of *c*·*A*_m_·M.

For *b*·*d* > 1, MaxEff >*L*·*a*/(*L*·*a*+1), = synergy. With increasing values of *c*·*A*_m_·M above 1, the MaxEff increases towards a ceiling value of 100%, i.e., above *L*·*a*/(*L*·*a*+1) if *L*·*a* >> 1. For decreasing values of *c*·*A*_m_·M below 1, the MaxEff goes towards *L*·*a*/(*L*·*a*+1).

For *b* < 1, MaxEff <*L*·*a*/(*L*·*a*+1). With increasing values of *c*·*A*_m_·M above 1 the MaxEff reduces towards a ceiling value of *L*·*a*/(*L*·*a*+1) . For reducing values of *c*·*A*_m_·M below 1, the MaxEff increases towards *L*·*a*/(*L*·*a*+1).

More details on dependence of MaxEff-ATSM on parameter combination are listed in the table.

As mentioned above, for *b*·*c*·*d*·*A*_m_·M >> 1, and *c*·*A*_m_·M ≥ 1, MaxEff is always independent of the value of *c*·*A*_m_·M.

***MaxEff****for EXOM*:

MaxEffs for EXOM are as well as for ATSM dependent on *c*·*d*·*A*_m_·M. Further, for *c*·*A*_m_·M >> 1 when *d* >> 1, EXOM-MaxEff goes to 100%, while for *c*·*A*_m_·M >> 1 but with *c*·*d*·*A*_m_·M << 1, it is determined by *a*/(*a*+*c*·*A*_m_·M). When *c*·*A*_m_·M ≤ 1 and *c*·*d*·*A*_m_·M >> 1, EXOM-MaxEff goes to 1, while for *d* << 1, it goes to zero.

MaxEff in ATSM is dependent on *L*·*a* and *b*·*c*·*d*·*A*_m_·M, Table 3, while MaxEff in EXOM is dependent on *a* and *c*·*d*·*A*_m_·M. In comparison, EXOM-MaxEff demonstrates complete independence of *b*, which is somewhat inconsistent. The independence is due to the definition of parameter *d* (*β*) in EXOM, where an alloster only affects the efficacy of an orthoster with no reciprocity. Thus, synergy and mixed inhibition are different between ATSM and EXOM, since the MaxEff-ATSM has both parameter *b* and *d* involved while EXOM only depends on *d*.

As already indicated, more details on parameter influences on IntEff, enhancement, allo-agonism, allo-synergy, MaxEff, and mixed inhibition are given in comments to Table 3.

Ceiling effects of enhancement and allo-agonism by positive allosteric modulators (PAMs) are hallmarks and often detected in experiment [17,35-37]. These ceiling effects appear for *A*_m_·M > 1, panels A, D, G, J, M, Q, T, and X in Figure 5. Ceiling effects for competitive inhibition are determined by cooperative binding constant *c* < 1 and appears for *c*·*A*_m_·M > 10, and best seen for *b*·*d* = 1, panels C, F, I, L, P, S, V, and Z in Figure 5.

The ATSM was rejected as model for allo-competitive inhibition by gallamine at muscarinic subtype M2 receptors [20]. Meanwhile, both ATSM and EXOM can nicely simulate competitive inhibition with values of *c* low enough to keep the parameter products *b*·*c*·*d*·*A*_m_·M for ATSM and *c*·*d*·*A*_m_·M for EXOM less than 10, exemplified in Figure 5C and F.

Allo-synergy, seen in the presence of allosters as a lift in MaxEff above MaxEff for othosters alone, is now commonly described for agonistic-PAMs as well [5,8,25,36]. In ATSM, these characteristics of PAMs with MaxEff above maximal response for endogenous ligands alone may be simulated with values of *b* and *d* when their product is > 1, Figure 5M-N, while EXOM can simulate allo-synergy for *d* > 1, not shown. Mixed inhibition, appearing as values of MaxEff lower than MaxEff with orthosters alone in the presence of NAMs, including pure non-competitive inhibition, may be simulated for *b*·*d* < 1 in ATSM, Figure 5U, and for *d* < 1 in EXOM, Figure 5Y. Published examples of negative allosteric effects are now increasing as more interest is invested in development of NAMs [12,32,60].

In both allo-synergy and allo-inhibition, parameter *c*, as its value is lowered, will narrow the gap between MaxEff in the presence and absence of an alloster; compare panels M-P and panels T-Z in Figure 5.

The lack of effect of parameter *b* on MaxEff in EXOM clearly weakens the theory, even though additional details have been presented on the behaviour of EXOM [34]. A variant of EXOM has been developed with lumped parameters thus avoiding the problem of a missing effect of parameter *b* in MaxEff [24].

### Comparison of best-fit analyses to experimental data for ATSM and EXOM

Results from analysis of experimental data with ATSM and EXOM are listed in Table 4. Ideally parameters in a theory should manage to stay constant when the theory is fitted to different data sets of the same experimental concentration-response system; for instance at increasing alloster concentrations. Therefore, the more the ratios in Table 4 for each single parameter deviate from unity in the present analysis, the worse is its model’s credibility.

**Table 4:** **Parameter ratios from best**-**fits with ATSM and EXOM on three data sets**

**Model for analysis**	**Data-figure #**	**Parameters ratios from best fits to concentration-response curves for orthosters at three different concentrations of allosters**			
		***b***	***c***	***d***	***A***_**m**_
ATSM	1	4.9	3.4	3.2	1.8
EXOM		46	1.9	3.0	2.6
ATSM	2	2.8	97	11	15
EXOM *		50	17	3.0	84
ATSM	3	1.6	9.2	16	1.5
EXOM		26	35	33	3.8

Each single parameter ratio from best fits with ATSM or EXOM is adapted from analysis of three sets of data in the literature, data-figures 1 to 3, see last section in Methods for references. Each data set consists of four concentration-response curves, where one curve is an orthoster concentration-response curve without an alloster present and the three other curves are orthoster concentration-responses experimentally obtained at three different alloster concentrations.

Parameters *a* and *A*_s_ for both ATSM and EXOM were initially determined by model-fits to the basic orthoster concentration-response curves without an alloster present. Obtained values for *a* and *A*_s_ were inserted in the model equations, which were then use for fitting to experimental data of the parameters *b*, *c*, *d*, and *A*_m_ in the theories. Each number in the table is a ratio between best-fit values with the largest deviation between two of three results from fits for the single parameter to three concentration-response curves at different alloster concentrations.

* For responses indicating spontaneous activity as in data-figure 2, evaluation by EXOM theory was performed by assuming a level of 9% spontaneous activity, thus fitting the EXOM distribution equation to 91% activity for all three alloster concentrations, 0.03, 0.1, and 0.3 μM ([37]). For ATSM used on data-figure 2, spontaneous activity was implemented by setting *L*/(1 + *L*) = 0.09. For data-figures 1 and 3a value of 0.01 was selected for *L.*

For a more detailed explanation of how the presented parameter ratios are obtained, see last section in Methods.

Both ATSM and EXOM have problems with a convincing determination of parameters fitted to data in data-figure 2. However, ATSM still seems to give the best result based on an overall evaluation of ratios for all four parameters from the three data sets of data-figure 2, Table 4.

Although exponentiation in form of a Hill coefficient may also be invoked for both models, such exponentiation was omitted in the present analysis. Also, an interpretation and detailed discussion of the actually obtained parameter values are beyond the scope of this paper.

Thus, based on the ratios in Table 4, it may be concluded that ATSM seems to be better than EXOM at evaluating possible parameter values with a requirement of consistency when determined at 3 different alloster concentrations, since in general most of the ratios are closer to unity when employing the ATSM.

## Conclusion

In a beautiful review, non-mechanistic EXOM against mechanistic ATSM is debated and further contrasted with an empirical general description of synagic behaviour of allosters in different experimental setups [17]. When system information is limited, analyses of allosteric behaviour by operational, empirical and mathematical approaches as Hill’s exponentiation are still valid. Meanwhile, analysing systems of allosteric synagics as discussed here, the best description of allosteric effects is by Hall’s millennium milestone mechanical model [22] due to shortcomings of EXOM. Limitations of mechanistic models as the ATSM are given with its assumptions, which usually both exclude more than two binding sites and multi-steps or parallel pathways. The ATSM may still replace the EXOM as a phenomenological model by applying assumptions similar to those for EXOM. For the future, allosteric models should be developed based on ATSM and implicating multi-binding and diverse pathways of receptor activation when needed. Thus, instead of switching to non-mechanistic approaches as EXOM or reduce requirements for the basic TCM to analyse such systems [20,26], phenomenological or extended forms of the ATSM should be preferred (e.g., [25]).

## Abbreviations

ATSM: Allosteric two-state model;EXOM: Extended operational model;cTSM: Cyclic two-state model;BLM: The Black & Leff operational model;7TMRs /GPCRs: 7 transmembrane helix G protein-coupled receptors;TCM: Ternary-complex model;ATCM: Allosteric ternary-complex model;EC50: Apparent dissociation constant at 50% activity;IntEff: Initial efficacy;MaxEff: Maximal efficacy;PAMs and NAMs: Positive and negative allosteric modulators;QSAR: Quantitative structure-activity-relationship

## Competing interest

The author declares no conflicts of interest.

## Authors’ contribution

NB developed and wrote the MS.

## Pre-publication history

The pre-publication history for this paper can be accessed here:

http://www.biomedcentral.com/2050-6511/14/4/prepub

## References

[B1] Ledford H Drug buddies Nature 2011 474 433 434 10.1038/474433a 21697923

[B2] Macilwain C Pharmaceutical industry must take its medicine Nature 2011 470 141 10.1038/470141a 21307893

[B3] Scannell JW Blanckley A Boldon H Warrington B Diagnosing the decline in pharmaceutical R&D efficiency Nat Rev Drug Discov 2012 11 191 200 10.1038/nrd3681 22378269

[B4] Elsinghorst PW Härtig W Gündisch D Mohr K Tränkle C Gütschow M A hydrazide linker strategy for heterobivalent compounds as ortho- and allosteric ligands of acetylcholine-binding proteins Curr Top Med Chem 2011 11 2731 2748 10.2174/156802611798184427 22039876

[B5] Gao ZG Verzijl D Zweemer A Ye K Göblyös A Ijzerman AP Functionally biased modulation of A(3) adenosine receptor agonist efficacy and potency by imidazoquinolinamine allosteric enhancers Biochem Pharmacol 2011 82 658 668 10.1016/j.bcp.2011.06.017 21718691 PMC3152598

[B6] Jensen PC Thiele S Steen A Elder A Kolbeck R Ghosh S Reversed binding of a small molecule ligand in homologous chemokine receptors - differential role of extracellular loop 2 Br J Pharmacol 2012 166 258 275 10.1111/j.1476-5381.2011.01771.x 22050085 PMC3415653

[B7] Melancon BJ Hopkins CR Wood MR Emmitte KA Niswender CM Christopoulos A Allosteric modulation of seven transmembrane spanning receptors: theory, practice, and opportunities for central nervous system drug discovery J Med Chem 2012 55 1445 1464 10.1021/jm201139r 22148748 PMC3349997

[B8] Valant C Felder CC Sexton PM Christopoulos A Probe dependence in the allosteric modulation of a G protein-coupled receptor: Implications for detection and validation of allosteric ligand effects Mol Pharmacol 2012 81 41 52 10.1124/mol.111.074872 21989256

[B9] Audet M Lagacé M Silversides DW Bouvier M Protein-protein interactions monitored in cells from transgenic mice using bioluminescence resonance energy transfer FASEB J 2010 24 2829 2838 10.1096/fj.09-144816 20335229

[B10] Chung KY Rasmussen SG Liu T Li S Devree BT Chae PS Conformational changes in the G protein Gs induced by the ß2 adrenergic receptor Nature 2011 477 611 615 10.1038/nature10488 21956331 PMC3448949

[B11] Comps-Agrar L Kniazeff J Nørskov-Lauritsen L Maurel D Gassmann M Gregor N The oligomeric state sets GABA(B) receptor signalling efficacy EMBO J 2011 30 2336 2349 10.1038/emboj.2011.143 21552208 PMC3116278

[B12] Henderson BJ Orac CM Maciagiewicz I Bergmeier SC McKay DB 3D-QSAR and 3D-QSSR models of negative allosteric modulators facilitate the design of a novel selective antagonist of human a4ß2 neuronal nicotinic acetylcholine receptors Bioorg Med Chem Lett 2012 22 1797 1813 10.1016/j.bmcl.2011.11.051 22285942 PMC3274641

[B13] Nygaard R Valentin-Hansen L Mokrosinski J Frimurer TM Schwartz TW Conserved water-mediated hydrogen bond network between TM-I, -II, -VI, and -VII in 7TM receptor activation J Biol Chem 2010 285 19625 19636 10.1074/jbc.M110.106021 20395291 PMC2885241

[B14] Peeters MC Wisse LE Dinaj A Vroling B Vriend G Ijzerman AP The role of the second and third extracellular loops of the adenosine A1 receptor in activation and allosteric modulation Biochem Pharmacol 2012 84 76 87 10.1016/j.bcp.2012.03.008 22449615

[B15] Schelshorn DW Joly F Mutel S Hampe C Breton B Mutel V Lateral Allosterism in the Glucagon Receptor Family: GLP-1 Induces GPCR Heteromer Formation Mol Pharmacol 2012 81 309 318 10.1124/mol.111.074757 22108912

[B16] Van Eps N Preininger AM Alexander N Kaya AI Meier S Meiler J Interaction of a G protein with an activated receptor opens the interdomain interface in the alpha subunit Proc Natl Acad Sci USA 2011 108 9420 9424 10.1073/pnas.1105810108 21606326 PMC3111277

[B17] Keov P Sexton PM Christopoulos A Allosteric modulation of G protein-coupled receptors: a pharmacological perspective Neuropharmacology 2011 60 24 35 10.1016/j.neuropharm.2010.07.010 20637785

[B18] Bindslev N Drug-Acceptor Interactions. Modeling Theoretical Tools to Test and Evaluate Experimental Equilibrium Effects 2008 1 Stockholm: Co-Action Publishing

[B19] De Amici M Dallanoce C Holzgrabe U Tränkle C Mohr K Allosteric ligands for G protein-coupled receptors: a novel strategy with attractive therapeutic opportunities Med Res Rev 2010 30 463 549 19557759 10.1002/med.20166

[B20] Ehlert FJ Griffin MT Two-state models and the analysis of the allosteric effect of gallamine at the m2 muscarinic receptor J Pharmacol Exp Ther 2008 325 1039 1060 10.1124/jpet.108.136960 18305010

[B21] Gomes I Ijzerman AP Ye K Maillet EL Devi LA G protein-coupled receptor heteromerization: a role in allosteric modulation of ligand binding Mol Pharmacol 2011 79 1044 1052 10.1124/mol.110.070847 21415307 PMC3102551

[B22] Hall DA Modeling the functional effects of allosteric modulators at pharmacological receptors: an extension of the two-state model of receptor activation Mol Pharmacol 2000 58 1412 1423 11093781 10.1124/mol.58.6.1412

[B23] Jäger D Schmalenbach C Prilla S Schrobang J Kebig A Sennwitz M Allosteric small molecules unveil a role of an extracellular E2/transmembrane helix 7 junction for G protein-coupled receptor activation J Biol Chem 2007 30 34968 34976 17890226 10.1074/jbc.M705563200

[B24] Kenakin TP '7TM receptor allostery: putting numbers to shapeshifting proteins Trends Pharmacol Sci 2009 30 460 469 10.1016/j.tips.2009.06.007 19729207

[B25] Stahl E Elmslie G Ellis J Allosteric modulation of the M3 muscarinic receptor by amiodarone and N-ethylamiodarone: application of the four-ligand allosteric two-state model Mol Pharmacol 2011 80 378 388 10.1124/mol.111.072991 21602476 PMC3164337

[B26] Leach K Sexton PM Christopoulos A Allosteric GPCR modulators: taking advantage of permissive receptor pharmacology. Supplementary data Trends Pharmacol Sci 2007 28 382 389 10.1016/j.tips.2007.06.004 17629965

[B27] Black JW Leff P Operational models of pharmacological agonism Proc R Soc Lond B 1983 220 141 162 10.1098/rspb.1983.0093 6141562

[B28] Ross EM Maguire ME Sturgill TW Biltonen RL Gilman AG Relationship between the beta-adrenergic receptor and adenylate cyclase J Biol Chem 1977 252 5761 5775 195960

[B29] De Lean A Stadel JM Lefkowitz RJ A ternary complex model explains the agonist specific binding properties of the adenylate cyclase coupled beta- adrenergic receptor J Biol Chem 1980 255 7108 7117 6248546

[B30] Stockton JM Birdsall NJ Burgen AS Hulme EC Modification of the binding properties of muscarinic receptors by gallamine Mol Pharmacol 1983 23 551 557 6865905

[B31] Ehlert FJ Estimation of the affinities of allosteric ligands using radioligand binding and pharmacological null methods Mol Pharmacol 1988 33 187 194 2828914

[B32] Bradley SJ Langmead CJ Watson JM Challiss RA Quantitative analysis reveals multiple mechanisms of allosteric modulation of the mGlu5 receptor in rat astroglia Mol Pharmacol 2011 79 874 885 10.1124/mol.110.068882 21321061 PMC3082933

[B33] Canals M Lane JR Wen A Scammells PJ Sexton PM Christopoulos A A Monod-Wyman-Changeux mechanism can explain G protein-coupled receptor (GPCR) allosteric modulation J Biol Chem 2012 287 650 659 10.1074/jbc.M111.314278 22086918 PMC3249119

[B34] Kenakin TP Biased signaling and allosteric machines; new vistas and challenges for drug discovery Br J Pharmacol 2012 165 1659 1669 10.1111/j.1476-5381.2011.01749.x 22023017 PMC3372820

[B35] Leach K Davey AE Felder CC Sexton PM Christopoulos A The role of transmembrane domain 3 in the actions of orthosteric, allosteric, and atypical agonists of the M4 muscarinic acetylcholine receptor Mol Pharmacol 2011 79 855 865 10.1124/mol.111.070938 21300722

[B36] Smith NJ Ward RJ Stoddart LA Hudson BD Kostenis E Ulven T Extracellular loop 2 of the free fatty acid receptor 2 mediates allosterism of a phenylacetamide ago-allosteric modulator Mol Pharmacol 2011 80 163 173 10.1124/mol.110.070789 21498659 PMC3127537

[B37] Suratman S Leach K Sexton P Felder C Loiacono R Christopoulos A Impact of species variability and 'probe-dependence' on the detection and in vivo validation of allosteric modulation at the M4 muscarinic acetylcholine receptor Br J Pharmacol 2011 162 1659 1670 10.1111/j.1476-5381.2010.01184.x 21198541 PMC3057301

[B38] Wootten D Savage EE Valant C May LT Sloop KW Ficorilli J Allosteric modulation of endogenous metabolites as an avenue for drug discovery Mol Pharmacol 2012 82 281 290 10.1124/mol.112.079319 22576254

[B39] Birdsall NJ Class A GPCR heterodimers: evidence from binding studies Trends Pharmacol Sci 2010 31 499 508 10.1016/j.tips.2010.08.003 20870299

[B40] Jakubík J Janícková H El-Fakahany EE Doležal V Negative cooperativity in binding of muscarinic receptor agonists and GDP as a measure of agonist efficacy Br J Pharmacol 2011 162 1029 1044 10.1111/j.1476-5381.2010.01081.x 20958290 PMC3051377

[B41] Kiselyov VV Versteyhe S Gauguin L De Meyts P Harmonic oscillator model of the insulin and IGF1 receptors' allosteric binding and activation Mol Syst Biol 2009 5 1 12 10.1038/msb.2008.78 PMC2657531 19225456

[B42] Rovira X Roche D Serra J Kniazeff J Pin JP Giraldo J Modeling the binding and function of metabotropic glutamate receptors J Pharmacol Exp Ther 2008 325 443 456 10.1124/jpet.107.133967 18287211

[B43] Birnbaumer L Bearer CF Iyengar R A two-state model of an enzyme with an allosteric regulator site capable of metabolizing the regulatory ligand J Biol Chem 1980 255 3552 3557 6102566

[B44] Leff P The twostate model of receptor activation Trends Pharmacol Sci 1995 16 89 97 10.1016/S0165-6147(00)88989-0 7540781

[B45] Stephenson RP A modification of receptor theory Br J Pharmacol 1956 11 379 393 10.1111/j.1476-5381.1956.tb00006.x PMC1510558 13383117

[B46] Changeux JP Allosteric proteins: from regulatory enzymes to receptors - personal recollections Bioessays 1993 15 625 634 10.1002/bies.950150909 8240316

[B47] Monod J Wyman J Changeux J-P On the nature of allosteric transitions: a plausible model J Mol Biol 1965 12 88 118 10.1016/S0022-2836(65)80285-6 14343300

[B48] Changeux JP 50th anniversary of the word "allosteric" Protein Sci 2011 20 1119 1124 10.1002/pro.658 21574197 PMC3149185

[B49] Koshland DE Jr Application of a theory of enzyme specificity to protein synthesis Proc Natl Acad Sci 1958 44 98 104 10.1073/pnas.44.2.98 16590179 PMC335371

[B50] Katz B Thesleff S A study of the desensitization produced by acetylcholine at the motor end-plate J Physiol 1957 138 63 80 13463799 10.1113/jphysiol.1957.sp005838 PMC1363030

[B51] Furchgott RF Receptor mechanisms Ann Rev Pharmcol 1964 4 21 50 10.1146/annurev.pa.04.040164.000321

[B52] Furchgott RF The use of β-haloalkylamines in the differentiation of receptors and in the determination of dissociation constants of receptor-agonist complexes Adv Drug Res 1966 3 21 55

[B53] Kenakin TP Beek D Is prenalterol (H133/80) really a selective beta 1 adrenoceptor agonist? Tissue selectivity resulting from differences in stimulus–response relationships J Pharmacol Exp Ther 1980 213 406 413 6102602

[B54] Slack RJ Hall DA Development of operational models of receptor activation including constitutive receptor activity and their use to determine the efficacy of the chemokine TARC at the CC-chemokine receptor CCR4 Br J Pharmacol 2012 166 1774 1792 10.1111/j.1476-5381.2012.01901.x 22335621 PMC3402803

[B55] Ehlert FJ Suga H Griffin MT Analysis of agonism and inverse agonism in functional assays with constitutive activity: estimation of orthosteric ligand affinity constants for active and inactive receptor states J Pharmacol Exp Ther 2011 338 671 686 10.1124/jpet.111.179309 21576379 PMC3141894

[B56] Perdona E Costantini VJ Tessari M Martinelli P Carignani C Valerio E In vitro and in vivo characterization of the novel GABAB receptor positive allosteric modulator, 2-{1-[2-(4-chlorophenyl)-5-methylpyrazolo[1,5-a]pyrimidin-7-yl]-2-piperidinyl}ethanol (CMPPE) Neuropharmacology 2011 61 957 966 10.1016/j.neuropharm.2011.06.024 21756923

[B57] Hall DA Langmead CJ Matching models to data: a receptor pharmacologist's guide Br J Pharmacol 2010 161 1276 1290 10.1111/j.1476-5381.2010.00879.x 20977467 PMC3000653

[B58] Segel IH Enzyme kinetics. Behavior and analysis of rapid equilibrium and steady-state enzyme systems 1975 New York: Wiley & Sons reissued 1993

[B59] Holst B Frimurer TM Mokrosinski J Halkjaer T Cullberg KB Underwood CR Overlapping binding site for the endogenous agonist, small-molecule agonists, and ago-allosteric modulators on the ghrelin receptor Mol Pharmacol 2009 75 44 59 10.1124/mol.108.049189 18923064

[B60] Mueller R Dawson ES Meiler J Rodriguez AL Chauder BA Bates BS Discovery of 2-(2-benzoxazoyl amino)-4-aryl-5-cyanopyrimidine as negative allosteric modulators (NAMs) of metabotropic glutamate receptor 5 (mGlu5): from an artificial neural network virtual screen to an in vivo tool compound ChemMedChem 2012 7 406 414 10.1002/cmdc.201100510 22267125 PMC3517057

